# Effects of Macromolecular Crowding on Human Adipose Stem Cell Culture in Fetal Bovine Serum, Human Serum, and Defined Xeno-Free/Serum-Free Conditions

**DOI:** 10.1155/2017/6909163

**Published:** 2017-03-30

**Authors:** Mimmi Patrikoski, Michelle Hui Ching Lee, Laura Mäkinen, Xiu Min Ang, Bettina Mannerström, Michael Raghunath, Susanna Miettinen

**Affiliations:** ^1^Adult Stem Cell Group, BioMediTech, Faculty of Medicine and Life Sciences, University of Tampere, Tampere, Finland; ^2^Science Center, Tampere University Hospital, Tampere, Finland; ^3^NUS Tissue Engineering Program, Life Sciences Institute, National University of Singapore, Singapore 117510; ^4^Department of Biomedical Engineering, National University of Singapore, Singapore 117575; ^5^Department of Oral and Maxillofacial Diseases, University of Helsinki and Helsinki University Hospital, Helsinki, Finland; ^6^Department of Biochemistry, National University of Singapore, Singapore 117575; ^7^Institute of Chemistry and Biology, Zurich University of Applied Sciences (ZHAW), 8820 Wädenswil, Switzerland

## Abstract

Microenvironment plays an important role for stem cell proliferation and diﬀerentiation. Macromolecular crowding (MMC) was recently shown to assist stem cells in forming their own matrix microenvironment in vitro. The ability of MMC to support adipose stem cell (ASC) proliferation, metabolism, and multilineage diﬀerentiation was studied under diﬀerent conditions: fetal bovine serum- (FBS-) and human serum- (HS-) based media and xeno- and serum-free (XF/SF) media. Furthermore, the immunophenotype of ASCs under MMC was evaluated. The proliferative capacity of ASCs under MMC was attenuated in each condition. However, osteogenic diﬀerentiation was enhanced under MMC, shown by increased deposition of mineralized matrix in FBS and HS cultures. Likewise, signiﬁcantly greater lipid droplet accumulation and increased collagen IV deposition indicated enhanced adipogenesis under MMC in FBS and HS cultures. In contrast, chondrogenic diﬀerentiation was attenuated in ASCs expanded under MMC. The ASC immunophenotype was maintained under MMC with signiﬁcantly higher expression of CD54. However, MMC impaired metabolic activity and diﬀerentiation capacity of ASCs in XF/SF conditions. Both the supportive and inhibitory eﬀects of MMC on ASC are culture condition dependent. In the presence of serum, MMC maintains ASC immunophenotype and enhances adipogenic and osteogenic diﬀerentiation at the cost of reduced proliferation.

## 1. Introduction

Inside the human body, cells are surrounded by a microenvironment that is physiologically crowded with soluble factors, other cells, and extracellular matrix. The typical serum protein concentration of biological fluids are, for example, 30–70 g/L in interstitial fluid, 80 g/L in blood plasma, and even 200–350 g/L in cell cytoplasm [1]. In contrast, the typical in vitro serum protein concentration is 1–10 g/L, and the composition is maintained plain and simple with only the most essential components, for example, attachment and growth factors provided [1]. Thus, this poorly corresponds to the original tissue microenvironments. The macromolecular crowding (MMC) method addresses this question by modifying the microenvironment and facilitating the formation and remodeling of the extracellular matrix (ECM) [1, 2]. Macromolecular crowders function by way of the excluded volume effect (EVE) [3]. The volume that surrounds a given molecule becomes unavailable for other molecules, which leads to higher effective concentrations of different macromolecules in a solution. Furthermore, the amount of EVE is dependent on the fractional volume occupancy (FVO), which is defined as a fraction of the total volume occupied by macromolecules. Consequently, MMC influences many fundamentals, for example, rates of enzymatic reactions, formation of cytoskeleton, cell adhesion, and migration [1, 3–6].

Previously, the effect of MMC on stem cell behavior has mainly been studied using bone marrow-derived mesenchymal stem cells (BM-MSCs) in fetal bovine serum- (FBS-) based cultures [2, 7–9]. However, human adipose tissue is another attractive and abundant source of multipotent stem cells, known as the adipose stem cells (ASCs). ASCs have the ability to differentiate into several cell types of mesodermal origin [10, 11] and have low immunogenicity [12–14] and immunomodulatory properties [15–18]. Due to these characteristics, ASCs are promising candidates for clinical applications and several clinical trials are currently ongoing as reviewed [19, 20].

Stem cell therapies often require large numbers of cells and usually in vitro cell expansion preceding in vivo implantation. Traditionally, FBS has been used in stem cell cultures. For clinical purposes, animal-derived components in culture media raise safety concerns related to xenogeneic contaminations and transfer of xenogeneic infections upon cell transplantation [21, 22]. Also, antibodies against bovine antigens may be elicited by repeated administration of cells, which will affect the efficacy of cell-based treatments. Therefore, xeno-free (XF) alternatives such as human serum- (HS-) [23–25] or platelet- (PL-) derived supplements [26–28] as well as fully defined XF and serum-free (SF) conditions [29, 30] have been developed for ASC culture. HS-based approaches have not only potential for clinical use but also disadvantages, for example, limited availability and lot-to-lot variation [31]. These challenges can be avoided by replacing undefined and/or animal-derived components with defined XF/SF reagents that have a reproducible composition [29, 30]. Moreover, careful characterization of ASC behavior in clinically relevant in vitro culture conditions is important for the progress of cell-based therapies.

The aim of this study was to investigate the potential of MMC in supporting ASC proliferation and multilineage differentiation capacity and analyze the MMC effect on immunophenotype and morphology of ASCs in different culturing conditions considering clinical applications. In the current study, cell isolation and expansion were carried out in parallel in FBS- and HS-containing media and compared with those in defined XF/SF conditions. The MMC method has not been previously reported in ASC cultures and especially the use of different culture conditions under MMC is a novel approach. Our current study demonstrates both supportive and inhibitory aspects of the MMC on ASC cultures in different serum conditions and shows promising results of osteogenic and adipogenic differentiation of ASCs under MMC in FBS and HS cultures.

## 2. Materials and Methods

### 2.1. Ethical Considerations and Tissue Procurement

The collection of adipose tissue was approved by the ethics committee of the Pirkanmaa Hospital District in Tampere, Finland (ethical approval R03058). Adipose tissue samples were acquired during elective surgical procedures performed in the Department of Plastic Surgery, Tampere University Hospital, Tampere, Finland, with the patient's written consent, and the study was conducted in accordance with the Declaration of Helsinki 1975, revised in Hong Kong 1989. ASCs were isolated from adipose tissue samples that were obtained from four female donors (mean age 52 ± 12).

### 2.2. Isolation and Expansion of ASCs

ASCs were isolated from adipose tissue samples into three different culture conditions: medium containing FBS, HS, or defined XF/SF culture conditions. Isolation of ASCs was carried out using a mechanical and enzymatic method as described previously [10, 11, 30, 32]. Briefly, the adipose tissue was minced manually into small fragments and digested with collagenase NB 6 GMP Grade (SERVA Electrophoresis GmbH, Heidelberg, Germany, http://www.serva.de). The digested tissue was centrifuged and filtered in sequential steps to separate the ASCs from the surrounding tissue. For FBS and HS conditions, Dulbecco's modified Eagle's medium (DMEM)/F-12 1 : 1 (Life Technologies, Rockville, MD) was supplemented with 1% L-alanyl-L-glutamine (GlutaMAX I; Life Technologies), 1% antibiotics (p/s; 100 U/mL penicillin, 0.1 mg/mL streptomycin; Lonza, Walkersville, MD, http://www.lonza.com), and either 10% FBS (Life Technologies) or 10% HS (human serum type AB; Lonza). For XF/SF conditions, cells were isolated as described previously [30]. Briefly, after the isolation steps described previously, cells were seeded in carboxyl-coated flasks (PureCoat Carboxyl T75; BD Biosciences, Franklin Lakes, NJ, http://www.bdbiosciences.com) and expanded in StemPro® MSC SFM XenoFree (Life Technologies) supplemented with 1% GlutaMAX I, 0.3% antibiotics, and 10% StemPro MSC SFM XenoFree supplement. From passage 1 onwards, XF/SF cells were expanded in a StemPro MSC SFM XenoFree medium supplemented with CELLstart CTS coating (Life Technologies) according to the manufacturer's instructions. ASCs were detached in all conditions using TrypLE Select (Life Technologies). Culture media formulations used for FBS, HS, and XF/SF cultures are presented in Table 1. Cells from four donors were separately analyzed for immunophenotype, proliferation, metabolic activity, and differentiation toward osteogenic and chondrogenic lineage, while adipogenic differentiation was performed with three donor cells in FBS, HS, and XF/SF conditions.

### 2.3. Macromolecular Crowding in ASC Culture

**Figure 1:**
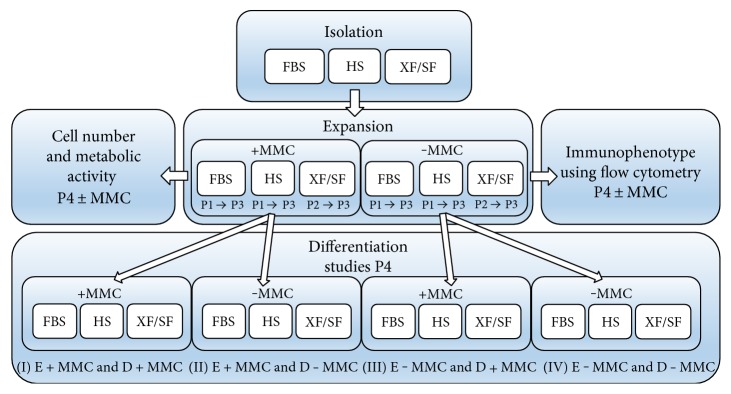
The workflow of the experiments under different culture conditions. The isolation of ASCs was carried out in parallel in FBS- and HS-containing media and in defined XF/SF conditions. After isolation from passage 1 onwards (FBS and HS conditions) and from passage 2 onwards (XF/SF condition), cells were divided into two populations. One half was expanded in standard media and the other half under MMC until the proliferation and differentiation analyses in passage 4. The osteogenic and adipogenic differentiation capacity of ASCs was studied in four different treatment groups (I–IV) and chondrogenic differentiation capacity in two treatment groups (II and IV). FBS, fetal bovine serum; HS, human serum; XF/SF, xeno-free/serum-free; MMC, macromolecular crowding; E ± MMC, expansion under macromolecular crowding/in standard medium; D ± MMC, differentiation under macromolecular crowding/in standard medium; P1, passage 1.

A cocktail of macromolecules containing FicollTM400 (PM400, 17-0300-50; GE Healthcare, Bio-Sciences AB) and FicollTM70 (PM70, 17-0310-50; GE Healthcare, Bio-Sciences AB) was dissolved in culture media at room temperature with gentle agitation. Fractional volume occupancy of 17% (v/v) was achieved with concentrations 37.5 mg/mL of FicollTM70 and 25 mg/mL of FicollTM400 as described previously [3]. The MMC culture media was sterile filtered before use. From passage 1 onwards, the FBS- and HS-expanded cells were divided into two populations and one-half was expanded in standard media and the other half under MMC until the analyses. For technical reasons, the XF/SF-expanded cells were divided into two populations from passage 2 onwards and expanded in standard XF/SF media and under MMC until the analyses. The workflow of the experiments is illustrated in Figure 1.

### 2.4. Adipose Stem Cell Immunophenotype

ASCs expanded in standard conditions and under MMC in FBS, HS, and SF/XF were analyzed using flow cytometry in passage 4 (FACSAria; BD Biosciences, Erembodegem, Belgium) to determine the immunophenotype of the cells. Monoclonal antibodies (MAbs) against CD11a-allophycocyanin (APC), CD80-phycoerythrin (PE), CD86-PE, CD105-PE (R&D Systems Inc., Minneapolis, MN, USA), CD3 (PE), CD14-phycoerythrin-cyanine (PECy7), CD19-PECy7, CD45RO-APC, CD54-fluorescein isothiocyanate (FITC), CD73-PE, CD90-APC (BD Biosciences), CD34-APC, and HLA-DR-PE (ImmunoTools GmbH, Friesoythe, Germany) were used. Analysis was performed on 10000 cells per sample, and unstained cell samples were used to correct for background autofluorescence [32].

### 2.5. ASC Morphology, Metabolic Activity, and Proliferation

ASCs were observed by light microscopy to detect morphological changes during cell expansion in FBS, HS, and XF/SF conditions in standard media and under MMC. The metabolic activity of ASCs in the aforementioned conditions was assessed with Cell Counting Kit-8 (CCK-8) (Takara Bio Inc., Shiga, Japan) in passage 4. The assay is based on the cleavage of tetrazolium salts by mitochondrial dehydrogenase enzyme, which enables colorimetric assessment of cell proliferation and metabolic activity. ASCs were seeded in 48-well plates at a density of 2500 cells/cm^2^, and metabolic/mitochondrial activity was assessed at 1, 4, 7, and 11 days. In brief, at each time point, the cell culture medium was removed, and DPBS (Dulbecco's phosphate-buffered saline, Lonza, BioWhittaker, Verviers, Belgium) and CCK-8 reagent were added 10 : 1. The 48-well plate was incubated for 3 hours at 37°C, and the relative mitochondrial activity was measured in a microplate reader (Victor 1429 Multilabel Counter; Wallac; Turku, Finland) at 450 nm.

Cell numbers were quantified using the CyQUANT® cell proliferation assay kit (Molecular Probes, Invitrogen, Paisley, UK) in passage 4 as described previously [33]. Briefly, cells were lysed with 0.1% Triton-X 100 buffer (Sigma-Aldrich), and the supernatant was collected and stored at −80°C until the final analysis. Fluorescence signals were measured with a microplate reader at 480/520 nm. The metabolic activity detected by CCK-8 cell proliferation assay was normalized to the cell number that was quantified by the CyQUANT cell proliferation assay kit.

### 2.6. Trilineage Differentiation Potential

The osteogenic, adipogenic, and chondrogenic differentiation potential was evaluated in XF/SF conditions versus HS and FBS media in standard conditions and under MMC in passage 4. The osteogenic and adipogenic differentiation capacity of ASCs was analyzed in four different treatment groups (I–IV) that are illustrated in Figure 1 and the chondrogenic differentiation was studied in two different treatment groups (II and IV) (Figure 1). The culture media formulations used for differentiation assays are shown in Table 1 and the workflow of the experiments is illustrated in Figure 1.

For osteogenic differentiation, cells were seeded on 12-well plates at a density of 2.0 × 10^3^ cells/cm^2^ in expansion media and the induction was initiated 48 h after cell seeding. After 28 days of induction, the osteogenic differentiation was quantified with the Alizarin red S method as described previously [25]. Briefly, cells were fixed with 4% paraformaldehyde (PFA) and stained with 2% Alizarin red solution (Sigma-Aldrich; pH 4.2), followed by several washes with distilled water. Light microscopic cell imaging was used for qualitative analysis and 100 mM cetylpyridinium chloride extraction (3 h incubation; Sigma-Aldrich) was used for quantitative analysis. The dye intensity was determined at 540 nm with a microplate reader.

For adipogenic differentiation, ASCs were seeded on 24-well plates at an initial density of 1.05 × 10^4^ cells/cm^2^. Adipogenic differentiation was initiated when the cells reached confluence as described previously [2] using three cycles of 4 days of induction followed by 3 days of maintenance. After 21 days of differentiation, Nile red staining and quantitative adherent cytometry were used to assess the area of cytoplasmic lipid accumulation. In brief, cell cultures were rinsed with PBS, fixed in 4% formaldehyde and costained for 30 min with 5 *μ*g/mL of Nile red (N3013; Sigma) for cytoplasmic lipid droplets and 0.5 *μ*g/mL of DAPI (D3571, Molecular Probes®; Life Technologies) for nuclear DNA as previously described [34]. Adherent cytometry was performed according to a previously described protocol [2]. Briefly, cell images were acquired using 2× magnification with a cool-SNAP HQ camera attached to a Nikon TE2000 microscope (Nikon Instruments) and analyzed using the Metamorph Imaging System Software 6.3v3 (Molecular Devices). The extent of adipogenic differentiation was quantified by area of Nile red fluorescence and normalized to nuclei count. End data corresponded to the total area of lipid droplets present per well normalized to cell number (*μ*m^2^/nuclei).

Collagen IV (Col IV) staining was used to evaluate the deposition of ECM proteins under MMC after 21 days of adipogenic induction. Briefly, ASCs were fixed with 4% formaldehyde and blocked with 3% bovine serum albumin (BSA) in PBS for 1 hour. Immunofluorescence was carried out using primary antibody for Col IV (ab6586, 1 : 500, Abcam, Cambridge, UK) and incubated overnight at 4°C in 1% BSA in PBS. The secondary antibody used was a 594 goat antibody against rabbit (Alexa Fluor A11072, 1 : 400, Molecular Probes, Thermofisher Scientific). Images were captured with an IX71 inverted fluorescence microscope (Olympus).

The chondrogenic differentiation potential was assessed by micromass culture as described previously [32, 35–37]. Briefly, 8 × 10^4^ cells were seeded on a 24-well culture plate in a 10 *μ*L volume and spheroids were allowed to form within 3 hours prior to the addition of chondrogenic induction medium. After 21 days of chondrogenic induction, differentiation was confirmed using Alcian blue staining as described earlier [37]. Briefly, micromass cultures were fixed with 4% PFA and stored in 70% ethanol. Pellets were dehydrated, embedded in paraffin, and sectioned at 5 *μ*m thickness. The sections were stained with Alcian blue (pH 1.0) to verify the presence of sulfated glycosaminoglycans (GAGs) with Nuclear Fast Red solution (Biocare Medical, Concord, MA, USA) as a counterstain.

### 2.7. Statistical Analyses

Statistical analyses were performed with IBM SPSS software version 21 (IBM Corp., Armonk, NY, www.ibm.com). Since the data was not normally distributed, a nonparametric Mann-Whitney *U* test was used to analyze the effect of different culture conditions on cell proliferation rate, cell surface proteins, and differentiation potential. The *p* values were multiplied by the number of comparisons when multiple comparisons were performed. For proliferation (CyQUANT) and metabolic activity (CCK-8), the *p* value was multiplied by 42, and for quantitative AR and NR staining data, the *p* value was multiplied by 24 that was a total number of performed comparisons. The nonparametric Spearman correlation test was used to study correlation between DNA amounts and metabolic activity of the cells. The results were considered statistically significant when the *p* value was less than 0.05. The data are presented as mean ± standard deviation (SD).

## 3. Results

### 3.1. The Effect of MMC on Cell Surface Markers

**Figure 2:**
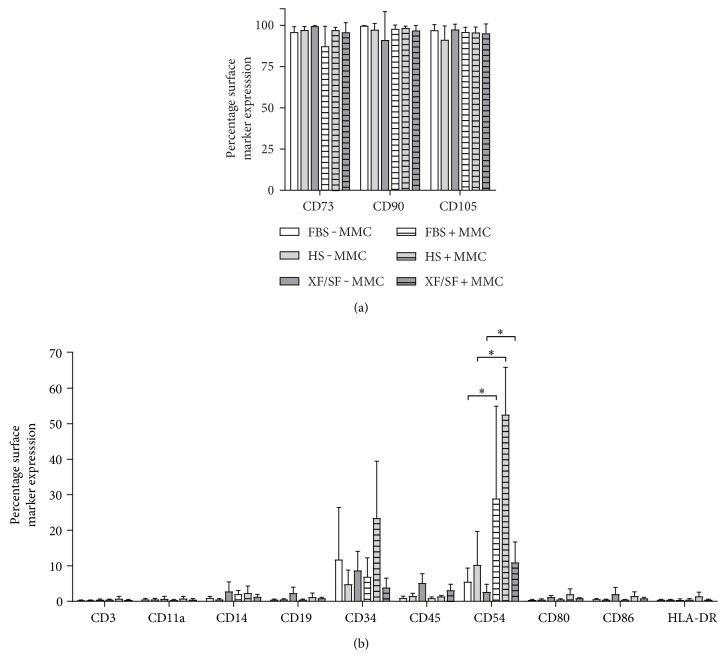
Surface marker expression of undifferentiated ASCs in different culture conditions FBS, HS, and XF/SF in standard conditions and under MMC. (a) The characteristic immunophenotype of ASCs was maintained under MMC- and ASC-expressed stem markers CD73, CD90, and CD105 in all studied conditions. (b) ASCs showed no expression of CD3, CD11a, CD14, CD19, CD80, CD86, and HLA-DR in all conditions. The expression of marker CD54 was significantly increased under MMC culture in every culture condition (*p* < 0.05). On average, ASCs showed low to moderate expression for hematopoietic progenitor stem cell marker CD34. Immunophenotype of ASCs was analyzed using flow cytometry at passage 3. Data are presented as mean ± SD. FBS, fetal bovine serum; HS, human serum; XF/SF, xeno-free/serum-free; MMC, macromolecular crowding.

The characteristic immunophenotype of ASCs was maintained under MMC- and ASC-expressed stem markers CD73, CD90, and CD105 in all studied conditions (Figure 2(a)). ASCs did not express CD3, CD11a, CD14, CD19, CD80, CD86, and HLA-DR in any of the studied conditions (Figure 2(b)). Interestingly, the expression of CD54 was significantly increased (*p* < 0.05) under MMC across all culture conditions. On average, ASCs showed low to moderate expression for hematopoietic progenitor stem cell marker CD34. Although some variation was observed between culture conditions, no statistical differences could be determined for CD34 due to large donor to donor variation. ASCs were exposed to MMC during cell expansion from passage 1 to 4 in FBS and HS media (Figure 1). However, MMC did not support the proliferation of XF/SF-expanded ASCs. To obtain sufficient cell number for the flow cytometric analysis, XF/SF cells were analyzed only after 7 days of exposure to MMC.

### 3.2. Cell Morphology

**Figure 3:**
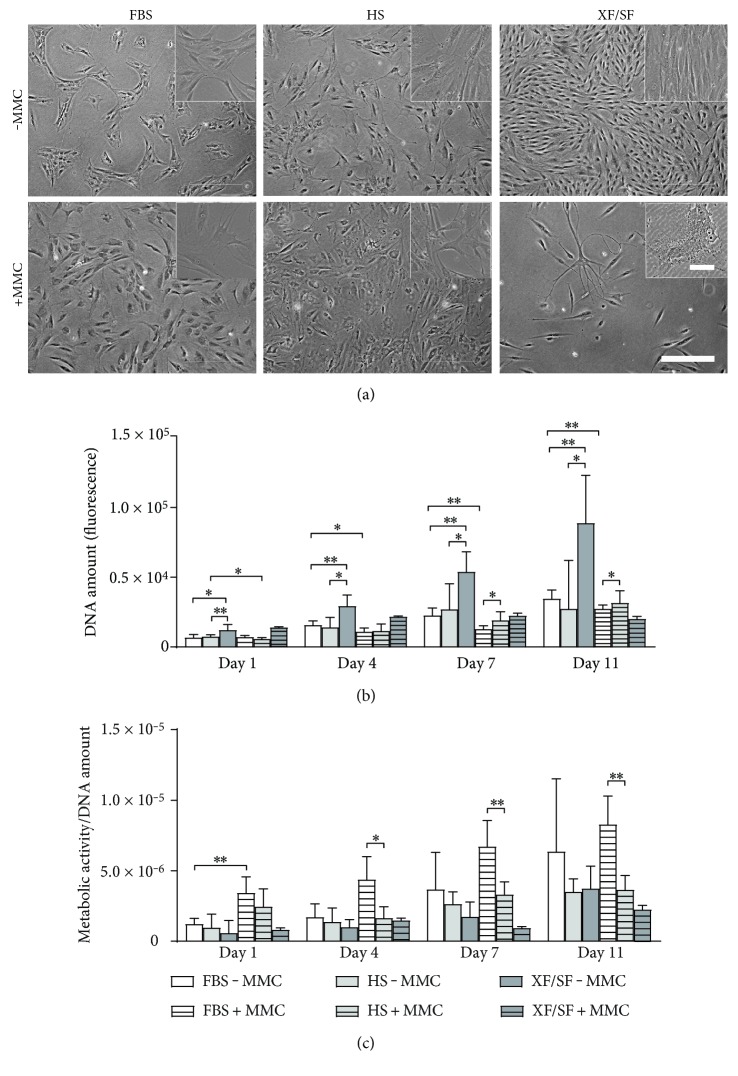
Morphology, cell number, and metabolic activity of ASCs under different culture conditions. (a) ASCs cultured under MMC adopted rounded morphology and became larger in size. The cell number under MMC in XF/SF cultures significantly decreased compared with that in standard cultures. Passage = 3. Scale bar 500 *μ*m (100 *μ*m in XF/SF inset). (b) The DNA amount indicating cell number in different culture conditions was analyzed with CyQUANT cell proliferation assay. Fluorescence signals were measured at 480/520 nm. A significantly higher cell number was observed in standard HS media at day 1 and in standard FBS media at days 4 and 7 and compared with that in MMC cultures. Under MMC, at time points—day 7 and 11—the cell number was significantly higher in HS media compared with that in FBS media. A significantly higher cell number was observed under standard cultures in XF/SF conditions compared with that in standard FBS and HS cultures at each time point—days 1, 4, 7, and 11. (c) The metabolic activity of ASCs was studied with CCK-8 assay and normalized to total DNA quantified by CyQUANT cell proliferation assay. Fluorescence signals were measured at 450 nm. A statistically significant increase in metabolic activity in FBS media under MMC was observed at day 1 compared with that in standard FBS media. ASCs in FBS media under MMC had a significantly higher metabolic activity compared with cells in HS media under MMC at days 4, 7, and 11 time points. Although a decreased cell number and metabolic activity was observed in XF/SF medium under MMC, no statistical differences could be established due to the low number donors that survived under MMC. ∗ indicates *p* < 0.05; ∗∗ indicates *p* < 0.001. Data are presented as mean ± SD. FBS, fetal bovine serum; HS, human serum; XF/SF, xeno-free/serum-free; MMC, macromolecular crowding.

The effect of MMC on cell morphology was evident in all studied culture conditions; cells adopted a rounded morphology and became larger in size under MMC. In particular, the morphology of XF/SF-expanded ASCs changed dramatically under MMC (Figure 3(a)). Cell numbers were dramatically decreased especially in XF/SF conditions under MMC compared with those in standard cultures. Cells with several extensions and multiple nuclei were also observed in XF/SF medium under MMC (Figure 3(a), inset).

### 3.3. Cell Number

ASCs were expanded under the presence or absence of MMC (±MMC) from passages 1 to 3 in FBS and HS media and from passages 2 to 4 in XF/SF media (Figure 1). The DNA amount indicating cell number (Figure 3(b)) was analyzed at passage 4. The significantly increased cell number was detected from day 1 to day 11 in each culture condition (Figure 3(b)), except in XF/SF medium under MMC (XF/SF + MMC). Moreover, a significantly higher cell number was observed under standard cultures in XF/SF conditions compared with that under standard FBS (*p* < 0.001) and HS (*p* < 0.05) cultures (XF/SF − MMC versus FBS − MMC and HS − MMC) at each time points—days 1, 4, 7, and 11. Cell numbers were also significantly increased in standard HS media (HS − MMC versus HS + MMC) at day 1 (*p* < 0.05) and in standard FBS media (FBS − MMC versus FBS + MMC) at days 4 (*p* < 0.05), 7, and 11 (*p* < 0.001) compared with those in MMC cultures. Under MMC, at time points—days 7 and 11—the cell number was significantly higher in HS media (*p* < 0.05) than in FBS media (HS + MMC versus FBS + MMC). The proliferation of ASCs in XF/SF media under MMC was studied with only one donor because the cells derived from the other three donors could not be expanded under MMC in XF/SF conditions. A decrease in cell number in XF/SF medium under MMC was evident compared with that in standard XF/SF culture (Figure 3(b)), but no statistical differences could be established due to the low number of repeats.

### 3.4. Metabolic Activity

The metabolic activity of ASCs was measured with CCK-8 assay and normalized to total DNA by CyQUANT cell proliferation assay (Figure 3(c)). A statistically significant increase in metabolic activity in FBS media under MMC (*p* < 0.001) was observed at day 1 compared with that in standard FBS media (FBS + MMC versus FBS − MMC) (Figure 3(c)). Under MMC, cells in FBS media had a significantly higher metabolic activity compared with cells in HS media (FBS + MMC versus HS + MMC) at days 4 (*p* < 0.001), 7, and 11 (*p* < 0.05) time points. Since MMC did not support the proliferation of ASCs in XF/SF media, the metabolic activity could only be studied with one donor in XF/SF conditions under MMC (XF/SF + MMC). The metabolic activity increased significantly (*p* < 0.001) over time (from day 1 to day 11) in each standard culture and under MMC in FBS media. The metabolic activity also correlated with the ASC number that was analyzed with the Spearman correlation test (coefficient 0.78; *p* < 0.001).

### 3.5. Multilineage Differentiation

**Figure 4:**
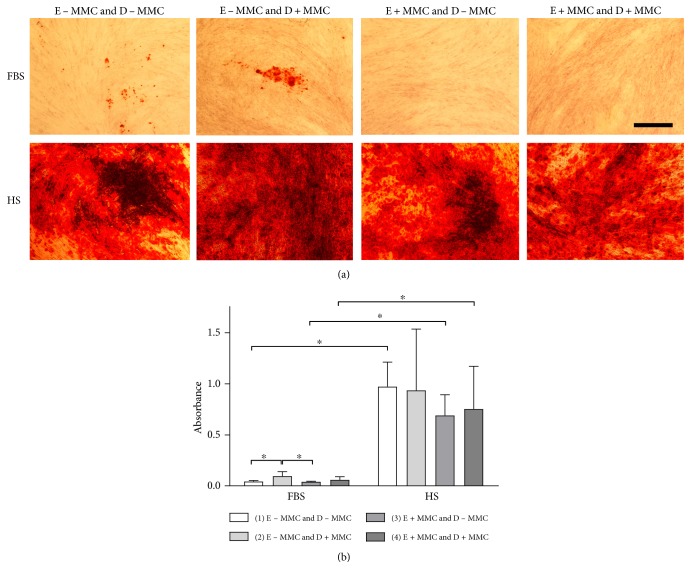
Alizarin red staining indicating the osteogenic differentiation potential of ASCs under different conditions. (a) Osteogenic differentiation was studied using quantitative Alizarin red staining after 28 days of osteogenic induction. (b) Alizarin red staining was quantified using cetylpyridinium chloride extraction. ASCs expanded in HS medium had stronger osteogenic differentiation capacity compared with those in FBS cultures. FBS cells expanded in standard medium and differentiated under MMC had a significantly stronger osteogenic differentiation capacity compared with cells expanded in standard or MMC cultures and differentiated in standard induction media. ASCs cultured in HS conditions had a strong osteogenic differentiation capacity in all studied conditions. Scale bar 500 *μ*m; ∗ indicates *p* < 0.05. Data are presented as mean ± SD. FBS, fetal bovine serum; HS, human serum; E ± MMC, expansion under macromolecular crowding/in standard medium; D ± MMC, differentiation under macromolecular crowding/in standard medium.

**Figure 5:**
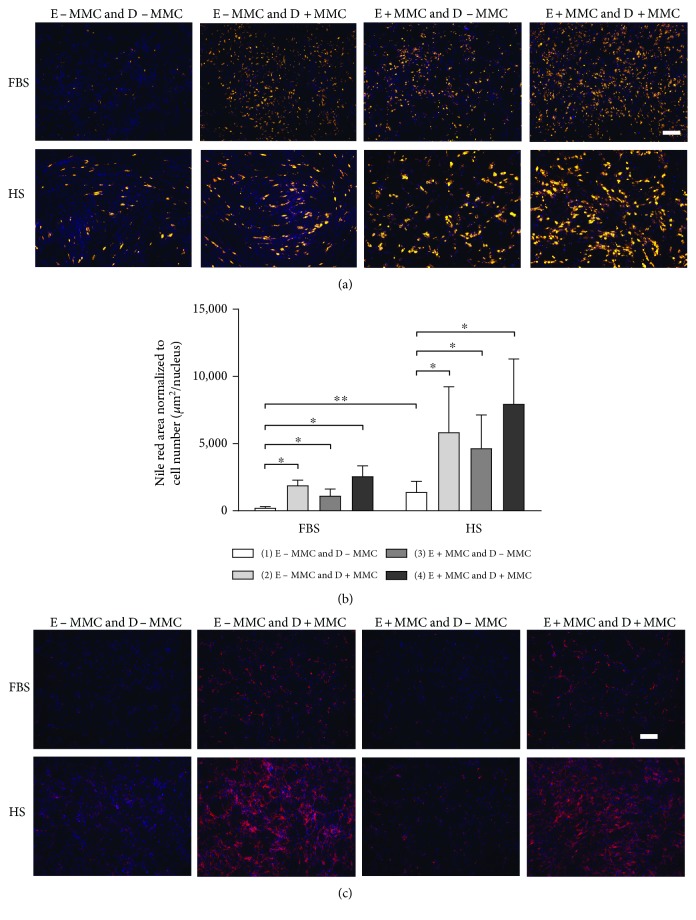
Quantitative Nile red staining indicating the adipogenic differentiation and collagen IV staining indicating adipogenic matrix deposition under different conditions. (a) The adipogenic differentiation was analyzed using Nile red staining that was normalized to cell number (b). In FBS and HS conditions, ASCs accumulated significantly more lipid content under induction with MMC compared with those under standard induction. A stronger differentiation was observed in HS media compared with that in FBS media in each induction group and significantly more lipid droplets were observed in standard HS induction media compared with those in standard FBS induction. Scale bar 500 *μ*m; ∗ indicates *p* < 0.05; ∗∗ indicates *p* < 0.001. Data are presented as mean ± SD. (c) Matrix deposition was evaluated using Col IV staining after adipogenic induction. Col IV deposition was enhanced under MMC induction compared with induction under MMC-free media in both FBS and HS conditions. Additionally, Col IV was more enhanced in HS media compared with that in FBS media based on qualitative analysis. Col IV deposition was in line with the results of lipid droplet accumulation that was confirmed using Nile red staining. Scale bar 500 *μ*m. FBS, fetal bovine serum; HS, human serum; E ± MMC, expansion under macromolecular crowding/in standard medium; D ± MMC, differentiation under macromolecular crowding/in standard medium.

**Figure 6:**
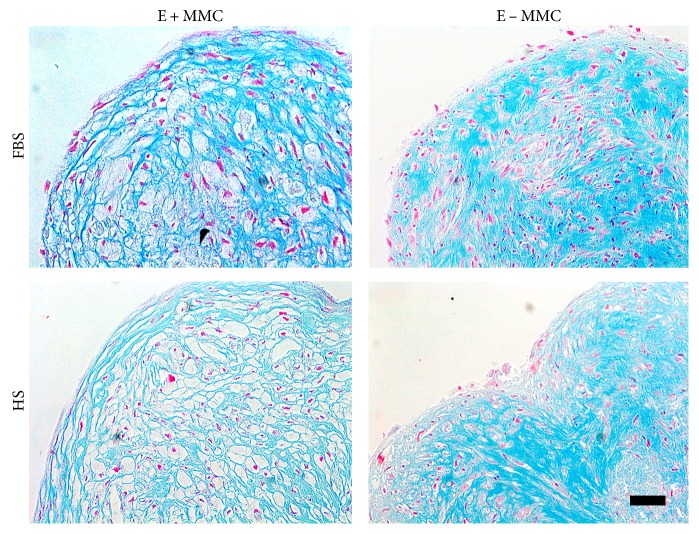
Alcian blue staining indicating the chondogenic differentiation of ASCs under different conditions. Qualitative Alcian blue staining suggested that proteoglycans were more deposited after expansion in standard conditions compared with expansion under MMC. A less dense histological architecture of the micromass pellet was observed on cells that were expanded under MMC prior to differentiation. Scale bar 50 *μ*m. FBS, fetal bovine serum; HS, human serum; E ± MMC, expansion under macromolecular crowding/in standard medium.

The osteogenic and adipogenic differentiation capacity was studied using standard induction protocols under the presence or absence of MMC (D ± MMC) after cell expansion in FBS, HS, and XF/SF conditions (E ± MMC) from passage 1 to 3 (HS and FBS conditions) and from passage 2 to 3 (XF/SF conditions) (Figure 1). Chondrogenic differentiation was studied using the standard induction only (D − MMC) after cell expansion under the presence or absence of MMC (D ± MMC). Osteogenic, adipogenic, and chondrogenic differentiation was induced at passage 4. Cells in XF/SF conditions under MMC did not show efficient differentiation capacities, which was in line with significantly reduced proliferation capacity observed under MMC in XF/SF media. Therefore, the results of XF/SF differentiations are presented in the supplemental data (see Supplementary Figure 1 in Supplementary Material available online at https://doi.org/10.1155/2017/6909163) and the multilineage differentiation results of FBS and HS cells are presented in Figures 4–6.

### 3.6. Osteogenic Differentiation

In FBS media, the addition of MMC during the induction phase significantly increased osteogenic differentiation (*p* < 0.05) in cells that had been expanded in MMC-free media (E − MMC and D + MMC versus E − MMC and D − MMC; Figure 4) analyzed using quantitative Alizarin red staining. Significantly increased osteogenic differentiation (*p* < 0.05) was observed in cells expanded in MMC-free media and induced under MMC compared with cells expanded under MMC and induced in MMC-free media in FBS condition (E − MMC and D + MMC versus E + MMC and D − MMC; Figure 4). Nevertheless, the HS conditions had a superior osteogenic differentiation (*p* < 0.05) compared with the FBS conditions (Figure 4). There were no statistical differences observed between different HS induction groups (Figure 4(b)), indicating that the addition of MMC had no significant effect on osteogenic differentiation in HS media. Significant osteogenic differentiation was observed in all HS and FBS conditions compared to their respective noninduced (negative control) cultures (Supplementary Figure 2).

### 3.7. Adipogenic Differentiation

The adipogenic differentiation was analyzed using a Nile red staining that was normalized to cell number. In FBS and HS conditions, ASCs that had been expanded in MMC-free media accumulated significantly more lipid content under induction with MMC (*p* < 0.05) compared with those in standard induction (E − MMC and D + MMC versus E − MMC and D − MMC; Figures 5(a) and 5(b)). Increased adipogenesis induced under MMC was also observed in ASCs that had been expanded in MMC media compared with that in standard induction (E + MMC and D + MMC versus E + MMC and D − MMC; Figures 5(a) and 5(b)) although not quantitatively significant. Interestingly, cells that had been expanded under MMC and induced under MMC-free media showed an increased capacity for adipogenesis (*p* < 0.05), compared with cells that were expanded in MMC-free media (E + MMC and D − MMC versus E − MMC and D − MMC; Figures 5(a) and 5(b)). Additionally, significantly more lipid droplets were observed in HS media (*p* < 0.001) compared with those in FBS media in standard inductions (HS E − MMC and D − MMC versus FBS E − MMC and D − MMC). Compared with that in noninduced (negative control) cultures of the same treatment group, significantly stronger adipogenic differentiation was observed in all conditions in FBS and HS media (Supplementary Figure 3).

MMC increased the deposition of matrix Col IV, which was evaluated using Col IV immunofluorescence staining after 21 days of adipogenic induction (Figure 5(c)). Col IV deposition was enhanced under MMC induction compared with that under standard adipogenic induction in both FBS and HS media (E ± MMC and D + MMC versus E ± MMC and D − MMC; Figure 5(c)). Additionally, Col IV deposition was enhanced in HS media compared with that in FBS media in all conditions, which was in line with the stronger adipogenic differentiation observed in HS media. Based on the qualitative Col IV staining, the amount of Col IV was dependent on the adipogenic differentiation efficiency. Also in XF/SF conditions, Col IV was more deposited under MMC induction compared with standard induction (E − MMC and D + MMC versus E − MMC and D − MMC; Supplementary Figure 1).

### 3.8. Chondrogenic Differentiation

Chondrogenic differentiation of ASCs was evident in all studied culture conditions. However, more proteoglycans were deposited after expansion in standard conditions based on qualitative Alcian blue staining. A less dense histological architecture of the micromass pellet was formed by the cells that were expanded under MMC prior to differentiation (Figure 6).

## 4. Discussion

Previous studies have demonstrated that MMC will increase the thermodynamic activities and biological processes by several orders of magnitude and will promote cells to recreate a more robust microenvironment in vitro [5, 38]. Our current study demonstrates the effect of MMC on ASC proliferation and differentiation under clinically relevant culture conditions. This is a novel approach and the influence of MMC on ASC characteristics has not been previously reported. However, we and others have shown that different culture conditions greatly influence ASC proliferation and differentiation capacity [24, 29, 30, 39]. As cell-based treatments are becoming more common, efficient in vitro cell expansion and differentiation methods are important to achieve an optimal and predictable clinical outcome.

In previous studies using human BM-MSCs, the benefits of MMC have been demonstrated by increased proliferation as well as efficient adipogenic differentiation [2, 3, 40]. Although most of the published studies have focused on the positive aspects of MMC, undesirable effects also exist such as protein destabilization and aggregate formation as recently reviewed [41]. Our current results demonstrate both positive and inhibitory effects of MMC on ASC behavior in culture from the viewpoint of expanding cells to reach therapeutically relevant numbers. Reduced proliferation capacity under MMC was observed in all studied culture conditions compared with that in standard cultures; on the other hand, strong differentiation capacity was detected under MMC (see next). The undesirable effects of MMC were obvious in XF/SF conditions as a significantly reduced proliferation capacity. In fact, cells expanded in standard XF/SF conditions had superior proliferation rates compared with cells cultured in standard FBS and HS cultures; however, proliferation of XF/SF cells was clearly reduced under MMC. Interestingly, ASCs expanded in FBS conditions under MMC showed increased metabolic activity compared with those in standard FBS cultures, whereas the metabolic activity in HS and XF/SF cultures was similar under MMC and in standard HS and XF/SF cultures.

Furthermore, changes in ASC morphology under MMC were evident in all culture conditions with cells adopting a larger size and more rounded shape. The major effect of MMC was observed in XF/SF cultures where cell morphology was changed under MMC. As cell number was significantly decreased in XF/SF conditions under a longer-term MMC exposure, the cells became large and round with many extensions with several nuclei observed occasionally. Similar observations have not been reported earlier as our study is the first to report use of MMC with XF/SF-cultured ASCs. The results showed that MMC method is not optimal for studied XF/SF cultures.

As the previous reports of increased proliferation rates under MMC were performed with BM-MSCs in FBS-containing media, this may explain the different outcomes. The MMC functions via EVE that is dependent on the FVO, which is defined as the fraction of the total volume occupied by macromolecules [3]. A use of a mixture of 70 and 400 kDa Ficoll as a crowding agent was first reported by Chen et al. who calculated the final concentration of macromolecules to a level corresponding to blood plasma protein concentrations that is approximately 80 mg/mL [3]. The FVO is calculated as optimal for BM-MSCs, and calculations are performed using the albumin concentrations of blood serum as a baseline. Thus, FVO should probably be further optimized for ASC cultures. Additionally, the effect of different serum conditions may change the equilibrium of the optimized FVO. As previously described, the basis for MMC function is its ability to support cells in recreating their own microenvironment in vitro [2, 3]. However, cells that produce naturally less ECM may not be induced to build the microenvironment even under MMC [3], which may apply to ASCs. Furthermore, it can be speculated that the ECM production is reduced in chemically defined XF/SF cultures hindering the suitability of MMC methods for XF/SF cells. Also, the weaker cell adherence that was speculated in XF/SF conditions [30] may interfere with the effects of MMC.

Due to low-metabolic activity of cells in XF/SF conditions under MMC, flow cytometric analysis was performed after a 7-day exposure to MMC. The immunophenotype of ASCs that were cultured in HS- and FBS-supplemented media was analyzed after culture from passage 1 to 3 under MMC. Overall, the characteristic immunophenotype of ASCs was maintained under MMC in all of the studied culture conditions. Low to moderate expression was observed for CD34 as reported previously [42, 43]. Based on our current results, the expression of CD34 appears to be more dependent on the culture media formulation than MMC exposure. Interestingly, the expression of CD54 was significantly higher under MMC in all of the studied culture conditions. CD54 is an intercellular adhesion molecule 1 (ICAM-1) that is typically expressed on endothelial cells and cells of the immune system and is involved in stabilizing cell-cell interactions, for example, in leukocyte and MSC interactions as previously reported [44]. However, more studies on cell-cell interactions and immunogenic properties of ASCs under MMC are required to demonstrate how increased CD54 expression translates to a specific function of ASCs.

In good agreement with previous studies, the ECM was found to be extensively deposited under MMC leading to more mature ECM [2, 3]. Under MMC, the intra- and extracellular proteins become aligned and the effect is maintained even in the absence of cellular interaction [1]. The ECM in turn affects cell-matrix interactions and promotes stronger cell adhesion, thus having an influence on the formation and structure of the cytoskeleton [1]. The current study demonstrates that the efficiency of adipogenic differentiation is in line with the deposition of matrix proteins, especially Col IV that is a major matrix component in adipocytes. This result indicates that a more mature state of ECM is produced under MMC. Additionally, culture condition (serum versus XF/SF condition) has an effect on the deposition of ECM proteins. In our study, more Col IV was deposited in HS media compared with that in FBS and XF/SF condition; however, the deposition of ECM proteins is likely associated with the more efficient differentiation that was observed in HS media.

While MMC exposure did not enhance ASC proliferation in the present study, a more efficient osteogenic and adipogenic differentiation was observed in FBS and HS cultures. In FBS conditions, cells had a significantly stronger osteogenic differentiation capacity under MMC induction compared with induction in standard conditions. Additionally, ASCs accumulated significantly more lipid droplets under MMC induction compared with induction under standard conditions. This result indicates the supportive influence of MMC on adipogenic and/or osteogenic commitment of ASCs in serum-containing medium. As previously demonstrated using BM-MSCs, crowding facilitates microenvironment formation and stabilizes or drives differentiation [2]. Ang et al. showed that the ECM was extensively remodeled toward a proadipogenic microenvironment under MMC in adipogenic induction media, which further promoted a strong adipogenic differentiation.

In contrast, our results showed that ASCs cultured in XF/SF conditions did not respond to MMC during differentiation induction. Significant differences in the intensity of osteogenic differentiation were also observed between HS and FBS media, with stronger osteogenic differentiation observed in HS medium compared with those in FBS medium. In line with this result, similar diminished osteogenic differentiation potential of ASCs cultured in FBS medium compared with HS medium has been reported previously by our group [25]. Our current results highlight the superior osteogenic potential of ASC-expanded HS-based medium compared to that of FBS conditions.

Both FBS and HS contain an undefined cocktail of growth factors and serum proteins and the composition varies between different serum lots. There are several studies on ASC culture in both FBS and HS, where lot-to-lot variation is evident [23–25, 39]. The majority of these studies suggest that cells expanded in HS medium show stronger osteogenic potential compared with cells expanded in FBS medium. We have previously shown that different culture conditions greatly influence proliferation and differentiation capacity of ASCs [30]. Thus, apart from the effects of MMC, cell viability, proliferation capacity, ECM composition, and trilineage differentiation potential are affected by a chosen culture condition. This effect was also evident in the current study.

Moreover, chondrogenic differentiation was studied in standard induction media after cell expansion both in standard media and under MMC. Based on qualitative Alcian blue staining, ASCs that were expanded in standard media had stronger capacity for chondrogenic differentiation compared with cells expanded under MMC, which was shown in stronger deposition of proteoglycans after standard cell expansion. The micromass pellet structure was also altered depending on culture condition, and cells expanded under MMC had less dense histological structure. The observed structural changes may be due to an increased remodeling activity under MMC as seen previously with adipogenic differentiation [2] which is based on matrix metalloproteinase activity. A less dense and more fibrous-like structure of the micromass pellet that was formed under MMC may not be specific for cartilage tissue; but on the other hand, the altered structure resembles a lacuna-like architecture that is typical for cartilage tissue. More studies should be performed to draw a conclusion on the crowding effects on chondrogenic differentiation potential of cells.

## 5. Conclusions

Careful characterization of ASC behavior with regard to clinically relevant in vitro culture conditions is highly important for the progress of cell-based therapies. In the current work, MMC was studied as an alternative method to facilitate ASC differentiation and proliferation. A strong capacity for osteogenic and adipogenic differentiation was observed for ASCs under MMC in serum-containing media, and thus, MMC is recommended to be used to enhance the differentiation capacity of ASC. Nevertheless, cell expansion should be performed in standard conditions. The ASC immunophenotype was maintained under MMC except the significantly increased expression of CD54. In conclusion, our results highlighted both supportive and inhibitory effects of MMC on ASC behavior and a success of MMC treatment appeared to be culture condition dependent. Still, novel methods to guide and strengthen ASC differentiation are desired and required. Our current study demonstrated that MMC is a potential technique to drive differentiation by modifying the ECM, which directly appears to modify cell morphology and to have an influence on the determination of a cell's fate.

## Supplementary Material

Supplementary Figure 1. The multilineage differentiation and Col IV deposition of XF/SF expanded ASCs The osteogenic, adipogenic and chondrogenic differentiation capacity of XF/SF cultured ASCs was studied in standard conditions and under MMC after cell expansion in both standard conditions and under MMC. After 28 days of inductions, osteogenic differentiation was studied using Alizarin Red (AR) staining, adipogenic differentiation using Nile Red (NR) staining and chondrogenic differentiation using Alcian Blue (AB) staining. Osteogenic differentiation was poor in XF/SF conditions under MMC; however, a cell sample from one donor survived in XF/SF conditions under MMC and showed efficient osteogenic differentiation after induction in standard osteogenic media. Adipogenic differentiation was not efficient in any of the studied conditions in XF/SF media, although a few lipid droplets were observed after cell expansion in standard XF/SF conditions. Moderate chondrogenic differentiation was observed in standard conditions and, similarly to FBS and HS cultures, an altered histological architecture of the micro mass pellet was observed after MMC expansion. Enhanced Col IV deposition under MMC induction was observed also in XF/SF conditions. Scale bar 500 μm (AR, NR, Col IV); 50 μm (AB). Abbreviations: E+/−MMC, expansion under macromolecular crowding/in standard medium; D+/−MMC, differentiation under macromolecular crowding/in standard medium; AR, Alizarin Red; NR, Nile Red; AB, Alcian blue; Col IV, collagen IV. Supplementary Figure 2. Quantitative Alizarin Red staining of ASCs Osteogenic differentiation was studied in osteogenic induction and control cultures using quantitative Alizarin Red staining and quantified with cetylpyridinium chloride extraction. ASC inducted in HS media had the strongest capacity for osteogenic differentiation compared with FBS and XF/SF induction. When compared with non-induced cultures of the same treatment group, ASCs in HS media had significantly stronger capacity for osteogenic differentiation in all induction groups. In FBS media, significantly stronger osteogenic differentiation was observed after expansion in standard medium and induction in either standard or MMC culture compared with control cultures of the same treatment group. The osteogenic differentiation capacity of ASCs in XF/SF conditions under MMC was poor. Only one donor cell sample showed capacity for osteogenic differentiation after expansion under MMC. The XF/SF cells that were expanded and differentiated in standard conditions showed variable potential for osteogenic differentiation. Due to large donor variation no statistical differences could be established for XF/SF cells. ∗ indicates p<0.05. Data are presented as mean ± SD. Abbreviations: E+/−MMC, expansion under macromolecular crowding/in standard medium; D+/−MMC, differentiation under macromolecular crowding/in standard medium. Supplementary Figure 3. Quantitative Nile Red staining of ASCs The adipogenic differentiation was analyzed in adipogenic induction and control cultures using Nile Red staining and normalized to cell number. ASCs differentiated in FBS and HS media had a significantly stronger capacity for adipogenic differentiation in all induction cultures compared with control cultures of the same treatment group. XF/SF cells did not show potential for adipogenic differentiation. ∗ indicates p<0.05; ∗∗ indicates p<0.001. Data are presented as mean ± SD. Abbreviations: E+/−MMC, expansion under macromolecular crowding/in standard medium; D+/−MMC, differentiation under macromolecular crowding/in standard medium.

## Figures and Tables

**Table 1:** Culture media formulations used for cell expansion and differentiation assays.

Medium	Basal media	Serum	Coating/coating-free supplements	Supplementation
Expansion FBS	DMEM/F-12 (Life Technologies, Gibco, Rockville, MD)	10% FBS (Life Technologies)	None	1% GlutaMAX (GlutaMAX I; Life Technologies), 1% p/s (p/s; 100 U/mL penicillin, 0.1 mg/mL streptomycin; Lonza)
Expansion HS	DMEM/F-12	10% HS (Lonza, Walkersville, MD)	None	1% GlutaMAX, 1% p/s
Expansion XF/SF	StemPro MSC SFM (Life Technologies)	None	CELLstart™ coating (Life Technologies)	1% GlutaMAX, 0,3% p/s, StemPro MSC SFM XenoFree supplement (Life Technologies)
Adipogenic FBS/HS	DMEM/F-12	10% FBS/HS	None	For 4 days induction during cyclic differentiation: 1% GlutaMAX, 1% p/s, 0.5 mM isobutylmethylxanthine (IBMX; Sigma), 0.2 mM indomethacin (Sigma), 1 *μ*M dexamethasone (Sigma), and 10 *μ*g/mL insulin (Sigma)
Adipogenic XF/SF	StemPro MSC SFM	None	CELLstart coating	For 4 days induction during cyclic differentiation: 1% GlutaMAX, 0,3% p/s, StemPro MSC SFM XenoFree supplement, same adipogenic supplements as in FBS/HS cultures
Osteogenic FBS/HS	DMEM/F-12	10% FBS/HS	None	1% GlutaMAX, 1% p/s, 150 *μ*M L-ascorbic acid 2-phosphate (Sigma), 10 mM beta-glycerophosphate (Sigma), 10 nM dexamethasone (Sigma)
Ostegenic XF/SF	StemPro MSC SFM	None	CELLstart coating	1% GlutaMAX, 0,3% p/s, StemPro MSC SFM XenoFree supplement, same osteogenic supplements as in FBS/HS cultures
Chondrogenic FBS/HS	DMEM/F-12	None	None	1% GlutaMAX, 0,2% p/s, 1x ITS + 1 (Sigma), 50 *μ*M L-ascorbic acid 2-phosphate, 55 *μ*M sodium pyruvate (Life Technologies), 23 *μ*M L-proline (Sigma), 10 ng/mL TGF-*β* (Sigma)
Chondrogenic XF/SF	StemPro MSC SFM	None	None	1% GlutaMAX, 0,2% p/s, StemPro MSC SFM XenoFree supplement, same chondrogenic supplements as in FBS/HS cultures

FBS: fetal bovine serum; HS: human serum; XF/SF: xeno-free/serum-free; StemPro MSC SFM XenoFree: StemPro mesenchymal stem cell serum-free and xeno-free media; p/s: penicillin and streptomycin; TGF-*β*: transforming growth factor beta.

## References

[1] Zeiger A. S., Loe F. C., Li R., Raghunath M., Van Vliet K. J. (2012). Macromolecular crowding directs extracellular matrix organization and mesenchymal stem cell behavior. *PLoS One*.

[2] Ang X. M., Lee M. H., Blocki A. (2014). Macromolecular crowding amplifies adipogenesis of human bone marrow-derived mesenchymal stem cells by enhancing the pro-adipogenic microenvironment. *Tissue Engineering Part A*.

[3] Chen C. Z., Loe F., Blocki A., Peng Y., Raghunath M. (2011). Applying macromolecular crowding to enhance extracellular matrix deposition and its remodeling in vitro for tissue engineering and cell-based therapies. *Advanced Drug Delivery Reviews*.

[4] Chen C. Z., Peng Y. X., Wang Z. B. (2009). The scar-in-a-jar: studying potential antifibrotic compounds from the epigenetic to extracellular level in a single well. *British Journal of Pharmacology*.

[5] Zhou H. X., Rivas G., Minton A. P. (2008). Macromolecular crowding and confinement: biochemical, biophysical, and potential physiological consequences. *Annual Review of Biophysics*.

[6] Ellis R. J. (2001). Macromolecular crowding: an important but neglected aspect of the intracellular environment. *Current Opinion in Structural Biology*.

[7] Dewavrin J. Y., Hamzavi N., Shim V. P., Raghunath M. (2014). Tuning the architecture of three-dimensional collagen hydrogels by physiological macromolecular crowding. *Acta Biomaterialia*.

[8] Satyam A., Kumar P., Fan X. (2014). Macromolecular crowding meets tissue engineering by self-assembly: a paradigm shift in regenerative medicine. *Advanced Materials*.

[9] Lee M. H., Goralczyk A. G., Kriszt R. (2016). ECM microenvironment unlocks brown adipogenic potential of adult human bone marrow-derived MSCs. *Scientific Reports*.

[10] Gimble J. M., Guilak F. (2003). Adipose-derived adult stem cells: isolation, characterization, and differentiation potential. *Cytotherapy*.

[11] Zuk P. A., Zhu M., Ashjian P. (2002). Human adipose tissue is a source of multipotent stem cells. *Molecular Biology of the Cell*.

[12] McIntosh K. R., Zvonic S., Garrett S. (2006). The immunogenicity of human adipose-derived cells: temporal changes in vitro. *Stem Cells*.

[13] Niemeyer P., Kornacker M., Mehlhorn A. (2007). Comparison of immunological properties of bone marrow stromal cells and adipose tissue-derived stem cells before and after osteogenic differentiation in vitro. *Tissue Engineering*.

[14] Ren M. L., Peng W., Yang Z. L. (2012). Allogeneic adipose-derived stem cells with low immunogenicity constructing tissue-engineered bone for repairing bone defects in pigs. *Cell Transplantation*.

[15] Puissant B., Barreau C., Bourin P. (2005). Immunomodulatory effect of human adipose tissue-derived adult stem cells: comparison with bone marrow mesenchymal stem cells. *British Journal of Haematology*.

[16] Kronsteiner B., Wolbank S., Peterbauer A. (2011). Human mesenchymal stem cells from adipose tissue and amnion influence T-cells depending on stimulation method and presence of other immune cells. *Stem Cells and Development*.

[17] Cui L., Yin S., Liu W., Li N., Zhang W., Cao Y. (2007). Expanded adipose-derived stem cells suppress mixed lymphocyte reaction by secretion of prostaglandin E2. *Tissue Engineering*.

[18] Crop M. J., Baan C. C., Korevaar S. S. (2010). Inflammatory conditions affect gene expression and function of human adipose tissue-derived mesenchymal stem cells. *Clinical and Experimental Immunology*.

[19] Casteilla L., Planat-Benard V., Laharrague P., Cousin B. (2011). Adipose-derived stromal cells: their identity and uses in clinical trials, an update. *World Journal of Stem Cells*.

[20] Lim M. H., Ong W. K., Sugii S. (2014). The current landscape of adipose-derived stem cells in clinical applications. *Expert Reviews in Molecular Medicine*.

[21] Selvaggi T. A., Walker R. E., Fleisher T. A. (1997). Development of antibodies to fetal calf serum with Arthus-like reactions in human immunodeficiency virus-infected patients given syngeneic lymphocyte infusions. *Blood*.

[22] Kadri N., Potiron N., Ouary M. (2007). Fetal calf serum-primed dendritic cells induce a strong anti-fetal calf serum immune response and diabetes protection in the non-obese diabetic mouse. *Immunology Letters*.

[23] Josh F., Kobe K., Tobita M. (2012). Accelerated and safe proliferation of human adipose-derived stem cells in medium supplemented with human serum. *Journal of Nippon Medical School*.

[24] Tateishi K., Ando W., Higuchi C. (2008). Comparison of human serum with fetal bovine serum for expansion and differentiation of human synovial MSC: potential feasibility for clinical applications. *Cell Transplantation*.

[25] Kyllonen L., Haimi S., Mannerstrom B. (2013). Effects of different serum conditions on osteogenic differentiation of human adipose stem cells in vitro. *Stem Cell Research & Therapy*.

[26] Trojahn Kolle S. F., Oliveri R. S., Glovinski P. V. (2013). Pooled human platelet lysate versus fetal bovine serum-investigating the proliferation rate, chromosome stability and angiogenic potential of human adipose tissue-derived stem cells intended for clinical use. *Cytotherapy*.

[27] Blande I. S., Bassaneze V., Lavini-Ramos C. (2009). Adipose tissue mesenchymal stem cell expansion in animal serum-free medium supplemented with autologous human platelet lysate. *Transfusion*.

[28] Naaijkens B. A., Niessen H. W., Prins H. J. (2012). Human platelet lysate as a fetal bovine serum substitute improves human adipose-derived stromal cell culture for future cardiac repair applications. *Cell and Tissue Research*.

[29] Al-Saqi S. H., Saliem M., Asikainen S. (2014). Defined serum-free media for in vitro expansion of adipose-derived mesenchymal stem cells. *Cytotherapy*.

[30] Patrikoski M., Juntunen M., Boucher S. (2013). Development of fully defined xeno-free culture system for the preparation and propagation of cell therapy-compliant human adipose stem cells. *Stem Cell Research & Therapy*.

[31] Parker A. M., Shang H., Khurgel M., Katz A. (2007). Low serum and serum-free culture of multipotential human adipose stem cells. *Cytotherapy*.

[32] Lindroos B., Boucher S., Chase L. (2009). Serum-free, xeno-free culture media maintain the proliferation rate and multipotentiality of adipose stem cells in vitro. *Cytotherapy*.

[33] Lindroos B., Maenpaa K., Ylikomi T., Oja H., Suuronen R., Miettinen S. (2008). Characterisation of human dental stem cells and buccal mucosa fibroblasts. *Biochemical and Biophysical Research Communications*.

[34] Greenspan P., Mayer E. P., Fowler S. D. (1985). Nile red: a selective fluorescent stain for intracellular lipid droplets. *The Journal of Cell Biology*.

[35] Zuk P. A., Zhu M., Mizuno H. (2001). Multilineage cells from human adipose tissue: implications for cell-based therapies. *Tissue Engineering*.

[36] Denker A. E., Nicoll S. B., Tuan R. S. (1995). Formation of cartilage-like spheroids by micromass cultures of murine C3H10T1/2 cells upon treatment with transforming growth factor-beta 1. *Differentiation*.

[37] Maenpaa K., Ella V., Mauno J. (2010). Use of adipose stem cells and polylactide discs for tissue engineering of the temporomandibular joint disc. *Journal of the Royal Society, Interface*.

[38] Kumar P., Satyam A., Fan X. (2015). Accelerated development of supramolecular corneal stromal-like assemblies from corneal fibroblasts in the presence of macromolecular crowders. *Tissue Engineering Part C, Methods*.

[39] Lindroos B., Aho K. L., Kuokkanen H. (2010). Differential gene expression in adipose stem cells cultured in allogeneic human serum versus fetal bovine serum. *Tissue Engineering Part A*.

[40] Rashid R., Lim N. S., Chee S. M., Png S. N., Wohland T., Raghunath M. (2014). Novel use for polyvinylpyrrolidone as a macromolecular crowder for enhanced extracellular matrix deposition and cell proliferation. *Tissue Engineering Part C, Methods*.

[41] Mittal S., Chowhan R. K., Singh L. R. (1850). Macromolecular crowding: macromolecules friend or foe. *Biochimica et Biophysica Acta*.

[42] Mirabet V., Solves P., Minana M. D. (2008). Human platelet lysate enhances the proliferative activity of cultured human fibroblast-like cells from different tissues. *Cell and Tissue Banking*.

[43] Rebelatto C. K., Aguiar A. M., Moretao M. P. (2008). Dissimilar differentiation of mesenchymal stem cells from bone marrow, umbilical cord blood, and adipose tissue. *Experimental Biology and Medicine (Maywood, N.J.)*.

[44] Ren G., Zhao X., Zhang L. (2010). Inflammatory cytokine-induced intercellular adhesion molecule-1 and vascular cell adhesion molecule-1 in mesenchymal stem cells are critical for immunosuppression. *Journal of Immunology*.

[45] Patrikoski M., Lee M. H., Mannerstrom B., Raghunath M., Miettinen S. (2016). Effects of macromolecular crowding on human adipose stem cell culture in fetal bovine serum, human serum and defined xeno-free/serum-free conditions. *European Cells & Materials*.

